# Septic Shock Caused by Coinfection of *Shewanella algae* Bloodstream Infection and Epstein‐Barr Virus: Clinical Characteristics and Genomic Analysis

**DOI:** 10.1002/mbo3.70221

**Published:** 2026-01-20

**Authors:** Jianmei Chen, Dong Ling, Feng Wang, Liping Liu, Yucheng Ren, Chengying Chen, Na Su

**Affiliations:** ^1^ Department of Clinical Laboratory Chongzhou People's Hospital Chongzhou China; ^2^ Department of Oncology and Hematology Chongzhou People's Hospital Chongzhou China

**Keywords:** coinfection, Epstein‐Barr virus, sepsis, *Shewanella algae*, whole‐genome sequencing

## Abstract

*Shewanella algae*, a marine‐origin opportunistic pathogen, has shown a significant increase in non‐coastal infections, yet its environmental adaptability and synergistic pathogenic mechanisms with Epstein‐Barr virus (EBV) coinfection remain unclear. This study reports a clinical case of *S. algae* bloodstream infection complicated by EBV reactivation leading to septic shock in Sichuan Province, China, and elucidates the molecular mechanisms through genomic analysis. Pathogen identification was performed via blood culture, antibiotic susceptibility testing, and genomic annotation. The strain harbored resistance genes (*acrB*, *tolC*, *tet(35)*, *golS*) and virulence factors (*bplL*/*bplF*, *clpC*/*clpP*, *tonB*). Phylogenetic analysis indicated the highest genetic affinity to freshwater‐derived *Shewanella chilikensis*, while pan‐genome analysis identified 1412 unique genes, including transmembrane transporters and carbohydrate‐active enzyme genes, suggesting freshwater adaptive evolution. Metagenomic next‐generation sequencing (mNGS) detected a high EBV load. The patient succumbed to multi‐organ failure. This study reveals that *S. algae* may evolve freshwater adaptability to cause inland infections, and EBV coinfection accelerates septic shock through immunosuppression and inflammatory cascades. Genomic analysis provides critical insights for precision diagnosis and treatment of polymicrobial infections.

## Graphical abstract

This study reports a septic shock case in inland China caused by coinfection of *Shewanella algae* bloodstream infection and Epstein‐Barr virus. Genomic analysis revealed freshwater adaptation of the strain via unique genes for transporters and carbohydrate‐active enzymes. EBV coinfection accelerated septic shock through immunosuppression and inflammatory cascades.

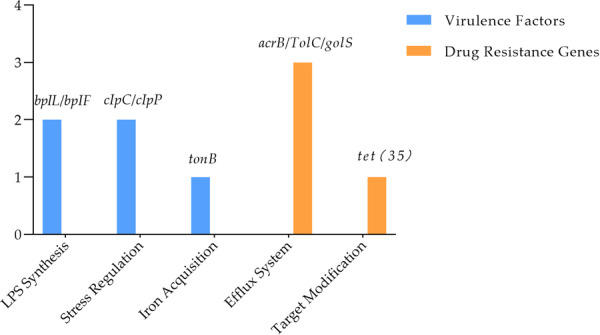

**Abbreviations**
AST: antimicrobial susceptibility testing
CARD: comprehensive antibiotic resistance database
CLSI: Clinical and Laboratory Standards Institute
EBV: Epstein‐Barr virus
IL6: interleukin‐6
mNGS: metagenomic next‐generation sequencing
PCT: procalcitonin
TCDB: transporter classification database
VFDB: virulence factor database

## 1 Introduction

*Shewanella algae* is a halophilic gram‐negative opportunistic pathogen traditionally associated with marine environments. Human infections are commonly linked to seawater exposure or immunocompromised states (Masmoudi et al. 2023; Ibrahim et al. 2021; Symanzik et al. 2022). In recent years, however, cases of infection in non‐coastal regions have been increasingly reported (Fernandes et al. 2023; Rodriguez‐Vargas et al. 2022; Weiss et al. 2021), suggesting its potential environmental adaptive evolution to occupy new ecological niches. Existing literature has documented mixed infections of *S. algae* with pathogens such as *Morganella morganii*, yet clinical studies on its coinfection with viruses, particularly the synergistic mechanisms in sepsis progression, remain unexplored (Huang et al. 2021; Martins Sousa et al. 2022). Notably, *S. algae* bloodstream infections carry a high mortality rate (35%–40%) (Ainoda et al. 2022) and often exhibit multidrug resistance (Wang et al. 2024; Huang et al. 2022), posing significant challenges to clinical management.

Epstein‐Barr virus (EBV), a ubiquitous human herpesvirus, can reactivate in immunocompromised patients (Houen and Trier 2020; Murata et al. 2021; Wei et al. 2023) and exacerbate inflammatory cascades by modulating host immune responses (Verbist and Nichols 2024; Xu et al. 2021; Ureshino et al. 2018). Studies indicate that EBV suppresses hepcidin expression (Mei et al. 2023), increasing serum free iron levels, while *S. algae* relies on iron acquisition systems (e.g., *tonB* gene) to hijack host iron resources (Liu et al. 2022; Fang et al. 2022). These interactions may form a metabolic symbiosis, further compromising host defenses. However, the molecular mechanisms underlying such cross‐species interactions in sepsis remain poorly understood.

This study presents the first clinical case of *S. algae* bloodstream infection co‐occurring with EBV reactivation in Sichuan Province, China. By integrating whole‐genome sequencing to dissect the strain's resistance profile, virulence factors, and evolutionary traits, we aim to provide empirical evidence for early diagnosis and treatment of inland *S. algae* infections. Our findings also highlight freshwater contamination as a potential novel transmission route, urging heightened vigilance in non‐coastal regions.

## 2 Materials and Methods

### 2.1 Case Report

An 80‐year‐old male patient was admitted on March 26, 2025, with complaints of “recurrent dizziness, palpitations, and dyspnea for 1 year, aggravated for 3 days.” His medical history included poorly controlled type 2 diabetes mellitus, megaloblastic anemia, and chronic cardiac insufficiency (preserved ejection fraction heart failure, cardiac function class III). The patient resided in a rural area and consumed unfiltered well water (prone to sewage contamination during rainy seasons), with no history of seawater or seafood exposure. Physical examination revealed high fever (39.4°C), anemic pallor (hemoglobin 37 g/L), and bilateral pitting edema. Laboratory tests showed elevated inflammatory markers (procalcitonin PCT 5.6 ng/mL, interleukin‐6 IL‐6 1232.1 pg/mL) and abnormal coagulation (D‐dimer 1.69 μg/mL). Chest CT indicated right pleural effusion with pulmonary atelectasis.

Blood culture turned positive after 26 h post‐admission, and the pathogen was identified as *S. algae* (strain H1, where H denotes host origin and 1 represents the strain number) using the VITEK 2 Compact system. Broth microdilution antimicrobial susceptibility testing revealed sensitivity to meropenem but resistance to ceftriaxone and ciprofloxacin (Table 1). Metagenomic next‐generation sequencing (mNGS) detected a high EBV DNA load (1.2 × 104 copies/mL; reference range: < 5 × 102 copies/mL). Serological tests confirmed EBV reactivation (EBV VCA‐IgM +, EA‐IgG ++ [Masmoudi et al. 2023]). Initial treatment included ceftriaxone (4 g QD), but the patient progressed to septic shock with multiple organ failure (acute liver failure, coagulopathy) within 72 h. Therapy was escalated to meropenem (1 g Q8H) combined with ganciclovir, alongside mechanical ventilation and closed thoracic drainage (800 mL bloody fluid evacuated).

**Table 1:** Antimicrobial susceptibility testing results of *Shewanella algae*.

Antibiotic class	Antibiotic name	MIC (μg/mL)	Interpretation (S/R)
β‐lactams	Ceftriaxone	64	R
β‐lactam/β‐lactamase inhibitor	Ampicillin/Sulbactam	2	S
	Cefoperazone/Sulbactam	4	S
Quinolones	Ciprofloxacin	4	R
	Levofloxacin	8	R
Sulfonamides + Trimethoprim	Trimethoprim/Sulfamethoxazole	0.5	S
Aminoglycosides	Amikacin	64	R
Carbapenems	Meropenem	0.25	S
	Imipenem	0.25	S
Monobactams	Aztreonam	32	R
Tetracyclines	Minocycline	0.25	S

*Note:* The MIC values were interpreted using CLSI M100 (2024) breakpoints. Please refer to Section 2 for details on the specific breakpoints applied for each antibiotic.

**Figure 1:**
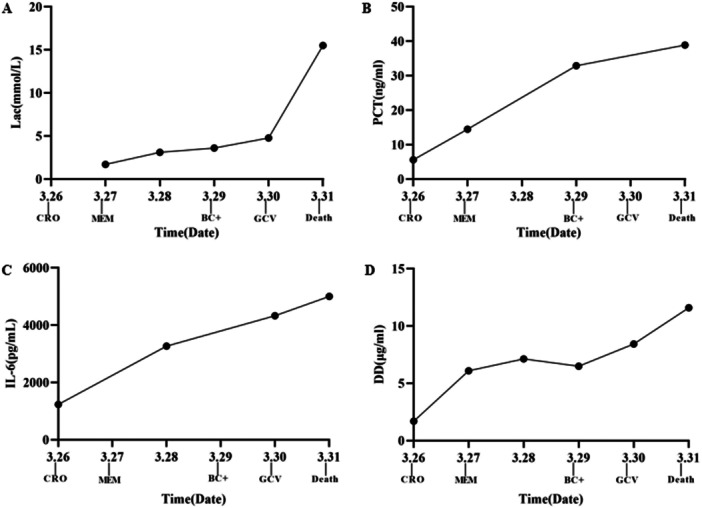
Dynamic changes in key laboratory parameters (March 26–31) and clinical interventions. (A) Lactate (Lac, mmol/L). (B) Procalcitonin (PCT, ng/mL). (C) Interleukin‐6 (IL‐6, pg/mL). (D) D‐dimer (DD, μg/mL). Labels on the X‐axis indicate the timing of key clinical events: CRO (ceftriaxone), BC+ (blood culture positive), MEM (meropenem), GCV (ganciclovir), and death.

The patient's condition deteriorated rapidly (Figure 1): PCT surged from 5.6 ng/mL (March 26) to 38.9 ng/mL (March 31), IL‐6 spiked to 5000 pg/mL, and organ failure progressed (peak ALT 842 U/L, creatinine 214.5 μmol/L, D‐dimer 11.6 μg/mL, hemoglobin 26 g/L). On March 31, the patient experienced cardiac arrest. Cardiopulmonary resuscitation restored transient spontaneous circulation, but irreversible multi‐organ failure led to clinical death at 18:26 after family withdrawal of life support.

### 2.2 Bacterial Pathogen Detection

Peripheral blood specimens were collected at admission and inoculated into BactAlert 3D aerobic and anaerobic blood culture bottles. Positive cultures were isolated and purified, followed by identification of the pathogen as *S. algae* using the VITEK 2 Compact system. For both the identification procedure and the antimicrobial susceptibility testing, *Pseudomonas aeruginosa* ATCC 27853 was used as the quality control strain. AST was performed using the broth microdilution method in accordance with the Clinical and Laboratory Standards Institute (CLSI) guidelines (M100, 2024). The minimum inhibitory concentration (MIC) values were determined for the antibiotics listed in Table 1. Due to the absence of specific CLSI breakpoints for *S. algae*, the MIC values were interpreted based on the criteria established for *P. aeruginosa* for the majority of antibiotics. However, for ceftriaxone, ampicillin/sulbactam, trimethoprim/sulfamethoxazole, and minocycline, for which *P. aeruginosa* breakpoints are not provided by CLSI, the interpretive criteria for *Escherichia coli* were applied.

### 2.3 Viral Pathogen Detection

Two milliliters of EDTA‐anticoagulated blood were collected from the patient. Plasma cell‐free DNA was extracted using the Plasma Free DNA Extraction Kit (TIANGEN DP338). After DNA library construction, sequencing was performed on the Illumina NextSeq. 550 platform. Raw sequencing data underwent quality control using FastQC (version 0.11.9) to remove low‐quality reads and adapter contamination. Clean reads were aligned to the NCBI NT/NR database (2025 version) for pathogen identification. EBV viral load was quantified by normalizing the number of mapped reads to copies per milliliter (copies/mL) using a standardized bioinformatics pipeline.

### 2.4 Genomic Sequencing and Bioinformatics Analysis

Following bacterial pure culture, genomic DNA was extracted using the TIANGEN DP338 DNA Extraction Kit. DNA concentration was quantified using Qubit 4.0 (≧20 ng/μL), and quality was assessed via agarose gel electrophoresis. Paired‐end sequencing (150 base pairs bp read length, total data volume 1.69 × 109 bp) was performed on the Illumina NextSeq. 550 platform. Raw sequencing data were processed with Trimmomatic v0.36 for quality control, including removal of low‐quality sequences and adapter contamination using a sliding window approach (Q20≧97.5%).

Genome assembly was conducted using SPAdes v3.5.0 to generate contigs. Gene annotation, including protein‐coding genes, rRNA, and tRNA, was performed using Prokka v1.10. Antibiotic resistance genes were annotated against the Comprehensive Antibiotic Resistance Database (CARD, https://card.mcmaster.ca/), while virulence factors were screened using the Virulence Factor Database (VFDB, http://www.mgc.ac.cn/VFs/).

### 2.5 Pan‐Genome and Functional Evolutionary Analysis

Pan‐genome analysis of the isolated strain (H1) and 30 closely related Shewanella strains from the NCBI RefSeq database was performed using Roary v3.13.0. Gene clusters were categorized as follows: core genes (shared by all strains), accessory genes (shared by≧2 strains), and unique genes (exclusive to H1). Metabolic functions of unique genes were annotated by aligning Prokka‐predicted protein sequences against the CAZy database (Carbohydrate‐Active Enzymes, http://www.cazy.org/) and the TCDB database (Transporter Classification Database, http://www.tcdb.org/) using HMMER3 v3.1b1. Key annotations included AA2 manganese peroxidases and ion transporters.

Phylogenetic analysis was conducted by aligning core single‐copy genes with MAFFT, followed by tree construction via FastTree using the neighbor‐joining method (Bootstrap values = 1000). Functional annotations were integrated to elucidate the freshwater adaptation mechanisms of the strain.

## 3 Results

### 3.1 Pathogen Detection Results

Blood culture turned positive after 26 h of incubation, with the pathogen identified as *S. algae*. mNGS detected a high EBV DNA load of 1.2 × 104 copies/mL (reference value: < 5 × 102 copies/mL). Serological tests confirmed EBV reactivation, with positive EBV VCA‐IgM (+) and elevated EA‐IgG (++).

### 3.2 Genomic Characteristics

**Figure 2:**
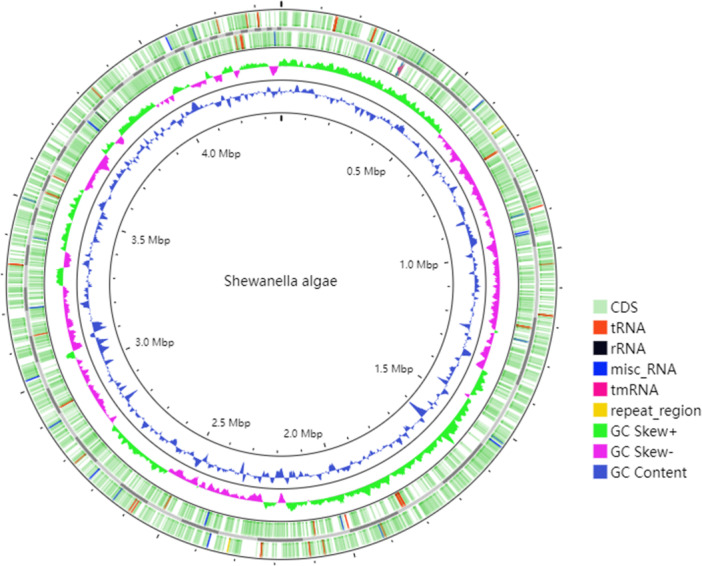
Circular genome map of *Shewanella algae* (H1). (Outer to inner rings: Distribution of coding regions and non‐coding RNAs, repetitive sequences, GC Skew [± values indicating the direction of replication strands], and dynamic GC content [10‐kb sliding window].)

The genome of the *S. algae* isolate (H1) had a total length of 4,445,496 base pairs (bp) with a GC content of 52.03%. The assembly comprised 90 contigs, with an N50 of 123,101 bp and a maximum contig length of 326,936 bp (Figure 2). Gene prediction revealed that coding regions accounted for 87.12% of the genome, encompassing 3948 protein‐coding genes, 6 rRNA genes, and 92 tRNA genes. Non‐coding regions were enriched with regulatory sequences and transposable elements.

### 3.3 Analysis of Resistance Gene Profile

**Figure 3:**
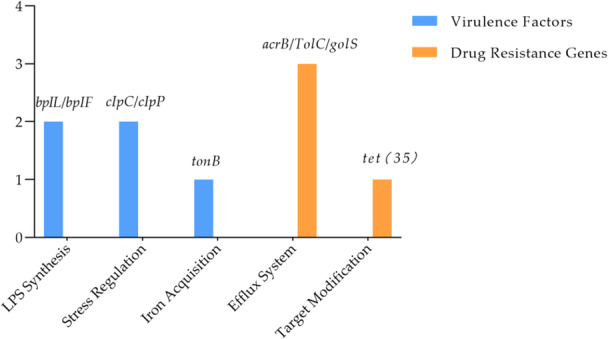
Antibiotic resistance genes (*acrB*, *tolC*, *tet(35)*, *golS*) and virulence factors (*bplL*/*bplF*, *clpC*/*clpP*, *tonB*) identified in *Shewanella algae* strain H1 via CARD and VFDB. Bar heights reflect relative gene abundance; colors distinguish resistance and virulence categories.

Annotation via the CARD database revealed four resistance‐associated genes in the *S. algae* (Figure 3):Efflux pump system genes: *acrB*(antibiotic efflux) and *TolC*(efflux channel protein), suggesting potential resistance to quinolones through active efflux mechanisms. Tetracycline resistance gene: *tet(35)*(ribosomal protection protein), likely mediating tetracycline resistance via target modification. Metal ion tolerance gene: *golS*, potentially involved in heavy metal resistance. AST demonstrated sensitivity to meropenem but resistance to quinolones (ciprofloxacin, levofloxacin) and ceftriaxone.

### 3.4 Virulence Factor Characteristics

Annotation via the VFDB database revealed that the strain harbored key virulence genes, including: Lipopolysaccharide synthesis‐related genes *bplL* and *bplF*, which may enhance bacterial endotoxin activity to promote host inflammatory responses. Stress response regulatory genes *clpC*/*clpP* (ATP‐dependent protease), involved in bacterial tolerance and survival within the host intracellular environment. Iron acquisition system gene *tonB* (siderophore transporter protein), hypothesized to exacerbate immunosuppression by competitively hijacking host iron resources (Figure 3).

### 3.5 Phylogenetic and Evolutionary Analysis

**Figure 4:**
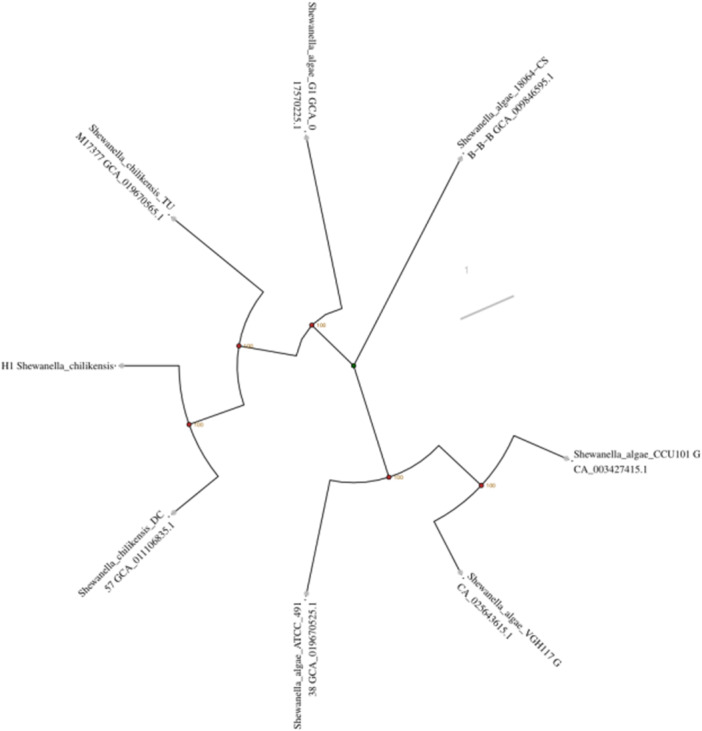
Phylogenetic tree constructed using core single‐copy genes (aligned with MAFFT and built via the neighbor‐joining method in FastTree, Bootstrap value = 1000). The H1 strain (red label) exhibits the closest genetic affinity to the freshwater‐derived *Shewanella chilikensis* (GCA_011106835.1).

**Figure 5:**
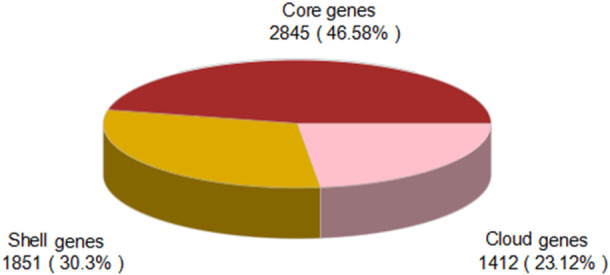
Pan‐genome composition analysis of *Shewanella algae* (H1).

Phylogenetic analysis based on 16S rRNA and core single‐copy genes revealed that the strain clustered with the freshwater‐derived *Shewanella chilikensis* (GCA_011106835.1) with the highest genetic affinity (Bootstrap value = 1000) (Figure 4). Pan‐genome analysis (including 30 closely related Shewanella strains) indicated that the H1 strain harbored 2845 core genes (46.58%) and 1412 unique genes (23.12%). Functional annotation of the unique genes revealed enrichment in transmembrane transporters (10.61% of TCDB‐annotated genes) and carbohydrate‐active enzymes (0.94% of CAZy‐annotated genes) (Figure 5), suggesting freshwater adaptive evolution through metabolic features such as transmembrane transporter gene enrichment.

## 4 Discussion

In this case, the patient had no history of seawater exposure, and the isolated strain (H1) exhibited the closest phylogenetic affinity to the freshwater‐derived *S. chilikensis*. Combined with the patient's long‐term consumption of contaminated well water, these findings suggest that *S. algae* may have colonized inland water systems through genomic adaptive evolution (Martín‐Rodríguez et al. 2022; Fu et al. 2024; Chen et al. 2020). Pan‐genome analysis revealed that the H1 strain harbored 1412 unique genes (23.12%) (Kim et al. 2020; Rosconi et al. 2022), enriched in transmembrane transporter genes (e.g.,*tonB*) and carbohydrate‐active enzyme (CAZy) genes. The *tonB* gene, encoding a siderophore transporter protein, may enhance bacterial tolerance to low‐salinity environments (Akinbosede et al. 2022; Pollet et al. 2021; Yong et al. 2023), while CAZy genes (e.g., AA2 manganese peroxidases) likely contribute to the degradation of complex organic matter in freshwater habitats (Nguyen et al. 2025; Vela Gurovic et al. 2021; Sun et al. 2023). These molecular insights provide evidence for the potential transmission of *S. algae* via agriculturally polluted inland water sources (Blanco and Díaz de Tuesta 2021).

Although AST indicated sensitivity to meropenem, the patient's treatment ultimately failed, suggesting that phenotypic results may underestimate the risk of cryptic resistance mechanisms (Mehrotra et al. 2023; Zhou et al. 2022). Genomic analysis revealed that efflux pump genes (*acrB* and *tolC*) mediate resistance to quinolones (e.g., ciprofloxacin) through active drug efflux (Dhara and Tripathi 2024; Albarri et al. 2022; Khoshnood et al. 2021). Notably, such efflux systems may exhibit low‐level activity against carbapenems (e.g., meropenem) (Lian et al. 2024; Tian et al. 2021), reducing intracellular drug concentrations and compromising clinical efficacy. Ceftriaxone resistance may arise from synergistic effects between penicillin‐binding protein (PBP) mutations and efflux pumps (Fischer et al. 2020; Sethuvel et al. 2023). Additionally, the metal ion tolerance gene *golS* might indirectly influence antibiotic susceptibility by regulating heavy metal resistance (Hasegawa et al. 2024). Severe immunosuppression caused by concurrent EBV reactivation likely further hindered bacterial clearance by antibiotics (Blot et al. 2022). These multifactorial interactions emphasize the need for genomic‐guided optimization of treatment regimens, such as administering high‐dose meropenem (1 g every 8 h) combined with efflux pump inhibitors (e.g., cilastatin).

The virulence genes carried by the strain exacerbate host pathological damage through multiple mechanisms. The lipopolysaccharide synthesis genes *bplL* and *bplF* significantly enhance bacterial endotoxin activity, activating the host TLR4/NF‐κB pathway and triggering a surge in inflammatory cytokines such as IL‐6 (Dong et al. 2020; Guo et al. 2024; Ali et al. 2024), directly leading to microcirculatory dysfunction. The stress response regulatory genes *clpC* and *clpP*, encoding an ATP‐dependent protease system, confer bacterial resistance to host oxidative stress, enabling survival within macrophage phagosomes and persistent release of toxic factors (Wen et al. 2025; Biswas et al. 2021). The iron acquisition gene *tonB* exacerbates anemia and suppresses neutrophil extracellular trap formation by competitively hijacking host free iron through the secretion of high‐affinity siderophores (Kumar et al. 2024). The dynamic elevation of lactate levels further corroborates the severity of microcirculatory impairment and tissue hypoxia (Yang et al. 2023, 2022). These metabolic disturbances synergize with bacterial endotoxins and EBV‐induced inflammatory storms, accelerating multi‐organ failure.

EBV reactivation reshapes the host immune response through multiple pathways. The EBV‐encoded latent membrane protein LMP1 downregulates the expression of host MHC class I molecules, impairing CD8 + T cell recognition efficiency of antigen presentation (Choi et al. 2021; Dudaniec et al. 2021), while simultaneously inhibiting the IFN‐γ signaling pathway, leading to exhaustion of specific immune responses. The EBV latent protein LMP2A activates the non‐canonical NF‐κB pathway, synergistically amplifying the secretion of pro‐inflammatory cytokines such as IL‐6 and TNF‐α with bacterial endotoxins, thereby forming an “inflammatory storm” that accelerates multi‐organ damage (Madayag et al. 2022; Yifei et al. 2024). Notably, the EBV‐encoded EBNA‐3C protein suppresses hepcidin expression, increasing serum free iron levels (Cortese et al. 2024). This establishes a metabolic mutualism network with the bacterial tonB‐mediated iron acquisition system, exacerbating anemia and immunosuppression (Li et al. 2021; Kloepfer and Kennedy 2023; Chevallereau et al. 2022).

Clinical Implications: Inland patients with unexplained sepsis, particularly those with comorbidities such as diabetes or anemia, should be vigilant against *S. algae* infection (Song et al. 2021). Early combination of blood culture, mNGS, and resistance gene detection is recommended (Sun et al. 2022). For treatment, carbapenems should be administered at full doses, and efflux pump inhibitors (e.g., cilastatin) may be added if necessary (Zack et al. 2024). In patients with EBV reactivation, combination therapy with ganciclovir and IL‐6 antagonists (e.g., tocilizumab) may be considered to block the inflammatory cascade.

## 5 Conclusions

*Shewanella algae* can cause infections in non‐coastal regions through freshwater adaptive evolution (e.g., enrichment of transmembrane transporters and CAZy genes). EBV coinfection accelerates septic shock progression via immune‐metabolic interactions. Clinically, pathogen surveillance in inland areas should be strengthened, and treatment strategies should be optimized through early integration of genomics and mNGS.

## Author Contributions

**Jianmei Chen:** conceptualization, methodology, validation, formal analysis, resources, data curation, writing – original draft, writing – review and editing, visualization, funding acquisition. **Dong Ling:** conceptualization, methodology, resources, writing – original draft. **Feng Wang:** conceptualization, supervision. **Liping Liu:** conceptualization, supervision. **Yucheng Ren:** methodology, project administration. **Chengying Chen:** methodology, resources, writing – review and editing, project administration. **Na Su:** validation, resources, funding acquisition.

## Ethics Statement

The authors have nothing to report.

## Consent

Written informed consent for publication was obtained from the patient's family.

## Conflicts of Interest

The authors declare no conflicts of interest.

## Data Availability

Data relating to this study are presented within the manuscript. Other materials are available from the corresponding author upon reasonable request.

## References

[mbo370221-bib-0001] Ainoda, Y. , E. Tanaka , T. Wajima , et al. 2022. “A Case of *Shewanella algae*‐Induced Bacteremia in Japan: Case Report and Literature Review.” Journal of Infection and Chemotherapy 28: 1430–1432. 10.1016/j.jiac.2022.06.015. 35777628

[mbo370221-bib-0002] Akinbosede, D. , R. Chizea , and S. A. Hare . 2022. “Pirates of the Haemoglobin.” Microbial Cell 9: 84–102. 10.15698/mic2022.04.775. 35434122 PMC8977872

[mbo370221-bib-0003] Albarri, O. , M. AlMatar , M. M. Öcal , and F. Köksal . 2022. “Overexpression of Efflux Pumps AcrAB and OqxAB Contributes to Ciprofloxacin Resistance in Clinical Isolates of *K. pneumonia* .” Current Protein & Peptide Science 23: 356–368. 10.2174/1389203723666220630162920. 35786184

[mbo370221-bib-0004] Ali, W. , K. Choe , J. S. Park , et al. 2024. “Kojic Acid Reverses LPS‐Induced Neuroinflammation and Cognitive Impairment by Regulating the TLR4/NF‐κB Signaling Pathway.” Frontiers in Pharmacology 15: 1443552. 10.3389/fphar.2024.1443552. 39185307 PMC11341365

[mbo370221-bib-0005] Biswas, S. , H. P. S. Dhaked , A. Keightley , and I. Biswas . 2021. “Involvement of ClpE ATPase in Physiology of *Streptococcus mutans* .” Microbiology Spectrum 9: e0163021. 10.1128/Spectrum.01630-21. 34851151 PMC8635124

[mbo370221-bib-0006] Blanco, G. , and J. A. Díaz de Tuesta . 2021. “Seasonal and Spatial Occurrence of Zoonotic *Salmonella* Serotypes in Griffon Vultures at Farmland Environments: Implications in Pathogen Pollution and Ecosystem Services and Disservices.” Science of the Total Environment 758: 143681. 10.1016/j.scitotenv.2020.143681. 33250252

[mbo370221-bib-0007] Blot, S. , E. Ruppé , S. Harbarth , et al. 2022. “Healthcare‐Associated Infections in Adult Intensive Care Unit Patients: Changes in Epidemiology, Diagnosis, Prevention and Contributions of New Technologies.” Intensive and Critical Care Nursing 70: 103227. 10.1016/j.iccn.2022.103227. 35249794 PMC8892223

[mbo370221-bib-0008] Chen, Y. J. , G. C. He , J. F. Cheng , et al. 2020. “Comparative Genomics Reveals Insights Into Characterization and Distribution of Quorum Sensing‐Related Genes in *Shewanella algae* From Marine Environment and Clinical Sources.” Comparative Immunology, Microbiology and Infectious Diseases 73: 101545. 10.1016/j.cimid.2020.101545. 32927298

[mbo370221-bib-0009] Chevallereau, A. , B. J. Pons , S. van Houte , and E. R. Westra . 2022. “Interactions Between Bacterial and Phage Communities in Natural Environments.” Nature Reviews Microbiology 20: 49–62. 10.1038/s41579-021-00602-y. 34373631

[mbo370221-bib-0010] Choi, I. K. , Z. Wang , Q. Ke , et al. 2021. “Mechanism of EBV Inducing Anti‐Tumour Immunity and Its Therapeutic Use.” Nature 590: 157–162. 10.1038/s41586-020-03075-w. 33361812 PMC7864874

[mbo370221-bib-0011] Cortese, M. , Y. Leng , K. Bjornevik , et al. 2024. “Serologic Response to the Epstein‐Barr Virus Peptidome and the Risk for Multiple Sclerosis.” JAMA Neurology 81: 515–524. 10.1001/jamaneurol.2024.0272. 38497939 PMC10949154

[mbo370221-bib-0012] Dhara, L. , and A. Tripathi . 2024. “Contribution of Genetic Factors Towards Cefotaxime and Ciprofloxacin Resistance Development Among Extended Spectrum Beta‐Lactamase Producing‐Quinolone Resistant Pathogenic Enterobacteriaceae.” Gene 893: 147921. 10.1016/j.gene.2023.147921. 37884102

[mbo370221-bib-0013] Dong, N. , X. Li , C. Xue , et al. 2020. “ *Astragalus polysaccharides* Alleviates LPS‐Induced Inflammation via the NF‐κB/MAPK Signaling Pathway.” Journal of Cellular Physiology 235: 5525–5540. 10.1002/jcp.29452. 32037545

[mbo370221-bib-0014] Dudaniec, K. , K. Westendorf , E. Nössner , and W. Uckert . 2021. “Generation of Epstein‐Barr Virus Antigen‐Specific T Cell Receptors Recognizing Immunodominant Epitopes of LMP1, LMP2A, and EBNA3C for Immunotherapy.” Human Gene Therapy 32: 919–935. 10.1089/hum.2020.283. 33798008

[mbo370221-bib-0015] Fang, L. , Y. Li , Y. Li , Y. Cao , and H. Song . 2022. “Transcriptome Analysis to Identify Crucial Genes for Reinforcing Flavins‐Mediated Extracellular Electron Transfer in *Shewanella oneidensis* .” Frontiers in Microbiology 13: 852527. 10.3389/fmicb.2022.852527. 35722328 PMC9198578

[mbo370221-bib-0016] Fernandes, S. , R. Sérvio , A. R. Silva , R. Tavares , and P. Rodrigues . 2023. “ *Shewanella algae*, an Emerging Human Pathogen: A Series of Four Cases From a Portuguese Hospital.” Cureus 15: e33686. 10.7759/cureus.33686. 36788829 PMC9920493

[mbo370221-bib-0017] Fischer, M. A. , S. Wamp , A. Fruth , F. Allerberger , A. Flieger , and S. Halbedel . 2020. “Population Structure‐Guided Profiling of Antibiotic Resistance Patterns in Clinical *Listeria monocytogenes* Isolates From Germany Identifies *pbpB3* Alleles Associated With Low Levels of Cephalosporin Resistance.” Emerging Microbes & Infections 9: 1804–1813. 10.1080/22221751.2020.1799722. 32691687 PMC7473133

[mbo370221-bib-0018] Fu, X. H. , H. X. Feng , Q. Huang , and W. Gou . 2024. “Pneumonia in an Elderly Tibetan Male Caused by *Shewanella algae*: A Case Report.” Medicine 103: e39197. 10.1097/md.0000000000039197. 39121328 PMC11315569

[mbo370221-bib-0019] Guo, S. , J. Zhang , Q. Zhang , et al. 2024. “ *Polygala tenuifolia* Willd. Extract Alleviates LPS‐Induced Acute Lung Injury in Rats via TLR4/NF‐κB Pathway and NLRP3 Inflammasome Suppression.” Phytomedicine 132: 155859. 10.1016/j.phymed.2024.155859. 38972239

[mbo370221-bib-0020] Hasegawa, L. A. , F. P. Vilela , and J. P. Falcão . 2024. “Antimicrobial Resistance, Virulence Potential and Genomic Epidemiology of Global Genomes of the Rare *Salmonella enterica* Serovar Orion.” Zoonoses and Public Health 71: 591–599. 10.1111/zph.13140. 38702905

[mbo370221-bib-0021] Houen, G. , and N. H. Trier . 2021. “Epstein‐Barr Virus and Systemic Autoimmune Diseases.” Frontiers in Immunology 11: 587380. 10.3389/fimmu.2020.587380. 33488588 PMC7817975

[mbo370221-bib-0022] Huang, W. H. , C. C. Kao , Y. C. Mao , et al. 2021. “ *Shewanella algae* and *Morganella morganii* Coinfection in Cobra‐Bite Wounds: A Genomic Analysis.” Life (Basel, Switzerland) 11: 329. 10.3390/life11040329. 33920102 PMC8069671

[mbo370221-bib-0023] Huang, Z. , K. Yu , S. Fu , Y. Xiao , Q. Wei , and D. Wang . 2022. “Genomic Analysis Reveals High Intra‐Species Diversity of *Shewanella algae* .” Microbial Genomics 8: 000786. 10.1099/mgen.0.000786. 35143386 PMC8942018

[mbo370221-bib-0024] Ibrahim, N. N. N. , N. M. Nasir , F. K. Sahrani , A. Ahmad , and F. Sairi . 2021. “Characterization of Putative Pathogenic *Shewanella algae* Isolated From Ballast Water.” Veterinary World 14: 678–688. 10.14202/vetworld.2021.678-688. 33935414 PMC8076470

[mbo370221-bib-0025] Khoshnood, S. , M. Heidary , A. Hashemi , et al. 2021. “Involvement of the AcrAB Efflux Pump in Ciprofloxacin Resistance in Clinical *Klebsiella pneumoniae* Isolates.” Infectious Disorders—Drug Targets 21: 564–571. 10.2174/1871526520999200905121220. 32888276

[mbo370221-bib-0026] Kim, Y. , C. Gu , H. U. Kim , and S. Y. Lee . 2020. “Current Status of Pan‐Genome Analysis for Pathogenic Bacteria.” Current Opinion in Biotechnology 63: 54–62. 10.1016/j.copbio.2019.12.001. 31891864

[mbo370221-bib-0027] Kloepfer, K. M. , and J. L. Kennedy . 2023. “Childhood Respiratory Viral Infections and the Microbiome.” Journal of Allergy and Clinical Immunology 152: 827–834. 10.1016/j.jaci.2023.08.008. 37607643 PMC10592030

[mbo370221-bib-0028] Kumar, A. , S. Chakravorty , T. Yang , T. A. Russo , S. M. Newton , and P. E. Klebba . 2024. “Siderophore‐Mediated Iron Acquisition by *Klebsiella pneumoniae* .” Journal of Bacteriology 206: e0002424. 10.1128/jb.00024-24. 38591913 PMC11112993

[mbo370221-bib-0029] Li, Z. J. , H. Y. Zhang , L. L. Ren , et al. 2021. “Etiological and Epidemiological Features of Acute Respiratory Infections in China.” Nature Communications 12: 5026. 10.1038/s41467-021-25120-6. PMC8373954 34408158

[mbo370221-bib-0030] Lian, S. , C. Liu , M. Cai , Y. Cao , and X. Xu . 2024. “Risk Factors and Molecular Characterization of Carbapenem Resistant *Escherichia coli* Recovered From a Tertiary Hospital in Fujian, China From 2021 to 2023.” BMC Microbiology 24: 374. 10.1186/s12866-024-03525-9. 39342086 PMC11438195

[mbo370221-bib-0031] Liu, L. , W. Wang , S. Wu , and H. Gao . 2022. “Recent Advances in the Siderophore Biology of *Shewanella* .” Frontiers in Microbiology 13: 823758. 10.3389/fmicb.2022.823758. 35250939 PMC8891985

[mbo370221-bib-0032] Madayag, K. , R. Incrocci , and M. Swanson‐Mungerson . 2022. “The Impact of Epstein‐Barr Virus Latent Membrane Protein 2A on the Production of B Cell Activating Factor of the Tumor Necrosis Factor Family (BAFF), APRIL and Their Receptors.” Immunity, Inflammation and Disease 10: e729. 10.1002/iid3.729. 36301035 PMC9597489

[mbo370221-bib-0033] Martín‐Rodríguez, A. J. , S. M. Higdon , K. Thorell , et al. 2022. “Comparative Genomics of Cyclic di‐GMP Metabolism and Chemosensory Pathways in *Shewanella algae* Strains: Novel Bacterial Sensory Domains and Functional Insights Into Lifestyle Regulation.” mSystems 7: e0151821. 10.1128/msystems.01518-21. 35311563 PMC9040814

[mbo370221-bib-0034] Martins Sousa, M. , M. von Hafe , A. Reis‐Melo , H. Silveira , G. Coutinho , and C. P. Moura . 2022. “Actinomyces and *Shewanella algae* Complicated Paediatric Mastoiditis: A Case Report of a Multidisciplinary Approach.” Access Microbiology 4: acmi000436. 10.1099/acmi.0.000436. 36644735 PMC9836059

[mbo370221-bib-0035] Masmoudi, S. , M. A. Khlif , H. Battikh , M. Zribi , M. Barsaoui , and K. Zitouna . 2023. “Case Report: *Shewanella algae*, a Rare Cause of Osteosynthesis‐Associated Infection.” F1000Research 12: 1465. 10.12688/f1000research.142096.2. 39239133 PMC11375409

[mbo370221-bib-0036] Mehrotra, T. , D. Konar , A. K. Pragasam , et al. 2023. “Antimicrobial Resistance Heterogeneity Among Multidrug‐Resistant Gram‐Negative Pathogens: Phenotypic, Genotypic, and Proteomic Analysis.” Proceedings of the National Academy of Sciences 120: e2305465120. 10.1073/pnas.2305465120. PMC10434301 37549252

[mbo370221-bib-0037] Mei, J. , X. Xiao , N. Liang , et al. 2023. “Clinical Significance of Serum Iron Metabolism‐Related Markers in Patients With Nasopharyngeal Carcinoma.” ORL 85: 223–230. 10.1159/000530714. 37311432

[mbo370221-bib-0038] Murata, T. , A. Sugimoto , T. Inagaki , et al. 2021. “Molecular Basis of Epstein‐Barr Virus Latency Establishment and Lytic Reactivation.” Viruses 13: 2344. 10.3390/v13122344. 34960613 PMC8706188

[mbo370221-bib-0039] Nguyen, T. V. , H. P. Trinh , and H. D. Park . 2025. “Genome‐Based Analysis Reveals Niche Differentiation Among *Firmicutes* in Full‐Scale Anaerobic Digestion Systems.” Bioresource Technology 418: 131993. 10.1016/j.biortech.2024.131993. 39694110

[mbo370221-bib-0040] Pollet, R. M. , L. M. Martin , and N. M. Koropatkin . 2021. “TonB‐Dependent Transporters in the Bacteroidetes: Unique Domain Structures and Potential Functions.” Molecular Microbiology 115: 490–501. 10.1111/mmi.14683. 33448497

[mbo370221-bib-0041] Rodriguez‐Vargas, C. M. , M. R. Pachar‐Flores , I. Espinosa , and E. Cherigo . 2022. “Surgical Site Infection by *Shewanella algae* After Major Surgery in a Patient With Colorectal Cancer: Case Report.” Cureus 14: e23158. 10.7759/cureus.23158. 35444888 PMC9009991

[mbo370221-bib-0042] Rosconi, F. , E. Rudmann , J. Li , et al. 2022. “A Bacterial Pan‐Genome Makes Gene Essentiality Strain‐Dependent and Evolvable.” Nature Microbiology 7: 1580–1592. 10.1038/s41564-022-01208-7. PMC9519441 36097170

[mbo370221-bib-0043] Sethuvel, D. P. M. , Y. D. Bakthavatchalam , M. Karthik , et al. 2023. “β‐Lactam Resistance in ESKAPE Pathogens Mediated Through Modifications in Penicillin‐Binding Proteins: An Overview.” Infectious Diseases and Therapy 12: 829–841. 10.1007/s40121-023-00771-8. 36877435 PMC10017896

[mbo370221-bib-0044] Song, J. E. , S. Kim , H. K. Kang , et al. 2021. “A Case of Bacterial Keratitis Caused by Multi‐Drug‐Resistant *Shewanella algae* Without Marine Exposure.” Oxford Medical Case Reports 2021: omab131. 10.1093/omcr/omab131. 34987857 PMC8713584

[mbo370221-bib-0045] Sun, C. C. , W. J. Zhao , W. Z. Yue , et al. 2023. “Polymeric Carbohydrates Utilization Separates Microbiomes Into Niches: Insights Into the Diversity of Microbial Carbohydrate‐Active Enzymes in the Inner Shelf of the Pearl River Estuary, China.” Frontiers in Microbiology 14: 1180321. 10.3389/fmicb.2023.1180321. 37425997 PMC10322874

[mbo370221-bib-0046] Sun, L. , S. Zhang , Z. Yang , et al. 2022. “Clinical Application and Influencing Factor Analysis of Metagenomic Next‐Generation Sequencing (mNGS) in ICU Patients With Sepsis.” Frontiers in Cellular and Infection Microbiology 12: 905132. 10.3389/fcimb.2022.905132. 35909965 PMC9326263

[mbo370221-bib-0047] Symanzik, C. , J. Esser , N. Pfennigwerth , C. Reuter , J. Bronnert , and M. Grade . 2022. “ *Shewanella algae* Bacteraemia in a Patient With a Chronic Ulcer After Contact With Seawater on Vacation in Turkey: A Case Report From a German Maximum‐Care Hospital.” New Microbes and New Infections 48: 101016. 10.1016/j.nmni.2022.101016. 36158312 PMC9490168

[mbo370221-bib-0048] Tian, Y. , Q. Zhang , L. Wen , and J. Chen . 2021. “Combined Effect of Polymyxin B and Tigecycline to Overcome Heteroresistance in Carbapenem‐Resistant *Klebsiella pneumoniae* .” Microbiology Spectrum 9: e0015221. 10.1128/Spectrum.00152-21. 34704782 PMC8549724

[mbo370221-bib-0049] Ureshino, H. , T. Ando , H. Kizuka , et al. 2018. “Tocilizumab for Severe Cytokine‐Release Syndrome After Haploidentical Donor Transplantation in a Patient With Refractory Epstein‐Barr Virus‐Positive Diffuse Large B‐Cell Lymphoma.” Hematological Oncology 36: 324–327. 10.1002/hon.2481. 28971493

[mbo370221-bib-0050] Vela Gurovic, M. S. , M. L. Díaz , C. A. Gallo , and J. Dietrich . 2021. “Phylogenomics, CAZyome and Core Secondary Metabolome of *Streptomyces albus* Species.” Molecular Genetics and Genomics 296: 1299–1311. 10.1007/s00438-021-01823-9. 34564766

[mbo370221-bib-0051] Verbist, K. , and K. E. Nichols . 2024. “Cytokine Storm Syndromes Associated With Epstein‐Barr Virus.” Advances in Experimental Medicine and Biology 1448: 227–248. 10.1007/978-3-031-59815-9_16. 39117818

[mbo370221-bib-0052] Wang, L. , S. Chen , M. Xing , et al. 2024. “Genome Characterization of *Shewanella algae* in Hainan Province, China.” Frontiers in Microbiology 15: 1474871. 10.3389/fmicb.2024.1474871. 39417074 PMC11480045

[mbo370221-bib-0053] Wei, H. T. , X. W. Xue , Q. Ling , P. Y. Wang , and W. X. Zhou . 2023. “Positive Correlation Between Latent Epstein‐Barr Virus Infection and Severity of Illness in Inflammatory Bowel Disease Patients.” World Journal of Gastrointestinal Surgery 15: 420–429. 10.4240/wjgs.v15.i3.420. 37032795 PMC10080598

[mbo370221-bib-0054] Weiss, T. J. , J. J. Barranco‐Trabi , A. Brown , T. T. Oommen , V. Mank , and C. Ryan . 2022. “Case Report: *Shewanella algae* Pneumonia and Bacteremia in an Elderly Male Living at a Long‐Term Care Facility.” American Journal of Tropical Medicine and Hygiene 106: 60–61. 10.4269/ajtmh.21-0614. PMC8733504 34781259

[mbo370221-bib-0055] Wen, Z. T. , K. Ellepola , and H. Wu . 2025. “MecA: A Multifunctional ClpP‐Dependent and Independent Regulator in Gram‐Positive Bacteria.” Molecular Microbiology 123: 433–438. 10.1111/mmi.15356. 40070161 PMC12121503

[mbo370221-bib-0056] Xu, X. J. , F. Y. Zhao , and Y. M. Tang . 2021. “Fulminant Cytokine Release Syndrome in a Paediatric Patient With Refractory Epstein‐Barr Virus‐Associated Haemophagocytic Lymphohistiocytosis Receiving Nivolumab Treatment.” Clinical Microbiology and Infection 27: 1710–1712. 10.1016/j.cmi.2021.08.015. 34425245

[mbo370221-bib-0057] Yang, L. , A. Gilbertsen , H. Xia , et al. 2023. “Hypoxia Enhances IPF Mesenchymal Progenitor Cell Fibrogenicity via the Lactate/GPR81/HIF1α Pathway.” JCI Insight 8: 163820. 10.1172/jci.insight.163820. PMC9977506 36656644

[mbo370221-bib-0058] Yang, X. , Y. Zhou , A. Liu , and Z. Pu . 2022. “Relationship Between Dynamic Changes of Microcirculation Flow, Tissue Perfusion Parameters, and Lactate Level and Mortality of Septic Shock in ICU.” Contrast Media & Molecular Imaging 2022: 1192902. 10.1155/2022/1192902. 36277595 PMC9568350

[mbo370221-bib-0059] Yifei, L. , Y. Jinjie , N. R. Beri , L. G. Roth , C. Ethel , and G. Benjamin E . 2024. “Germinal Center Cytokines Driven Epigenetic Control of Epstein‐Barr Virus Latency Gene Expression.” PLoS Pathogens 20: e1011939. 10.1101/2024.01.02.573986. 38683861 PMC11081508

[mbo370221-bib-0060] Yong, C. W. , B. Deng , L. M. Liu , X. W. Wang , and H. B. Jiang . 2023. “Diversity and Evolution of Iron Uptake Pathways in Marine Cyanobacteria From the Perspective of the Coastal Strain *Synechococcus* sp. Strain PCC 7002.” Applied and Environmental Microbiology 89: e0173222. 10.1128/aem.01732-22. 36533965 PMC9888192

[mbo370221-bib-0061] Zack, K. M. , T. Sorenson , and S. G. Joshi . 2024. “Types and Mechanisms of Efflux Pump Systems and the Potential of Efflux Pump Inhibitors in the Restoration of Antimicrobial Susceptibility, With a Special Reference to *Acinetobacter baumannii* .” Pathogens 13: 197. 10.3390/pathogens13030197. 38535540 PMC10974122

[mbo370221-bib-0062] Zhou, A. , S. Xie , H. Tang , et al. 2022. “The Dynamic of the Potential Pathogenic Bacteria, Antibiotic‐Resistant Bacteria, and Antibiotic Resistance Genes in the Water at Different Growth Stages of Grass Carp Pond.” Environmental Science and Pollution Research 29: 23806–23822. 10.1007/s11356-021-17578-0. 34817812

